# CD38 Inhibitor 78c Attenuates Pro-Inflammatory Cytokine Expression and Osteoclastogenesis in Macrophages

**DOI:** 10.3390/cells13231971

**Published:** 2024-11-28

**Authors:** William Lory, Nityananda Chowdhury, Bridgette Wellslager, Subramanya Pandruvada, Yan Huang, Özlem Yilmaz, Hong Yu

**Affiliations:** 1 Department of Biomedical and Community Health Sciences, College of Dental Medicine, Medical University of South Carolina, Charleston, SC 29425, USA; lory@musc.edu (W.L.); chowdhun@musc.edu (N.C.); bridgette.wellslager@nih.gov (B.W.); pandruv@musc.edu (S.P.); yilmaz@musc.edu (Ö.Y.); 2 Department of Endocrinology, College of Medicine, Medical University of South Carolina, Charleston, SC 29425, USA; huangyan@musc.edu

**Keywords:** CD38, NAD^+^, cytokine, osteoclast, podosome, bone loss

## Abstract

CD38, a nicotinamide adenine dinucleotide (NAD^+^) glycohydrolase, increases during infection or inflammation. Therefore, we aimed to evaluate the effects of a CD38 inhibitor (78c) on NAD^+^ levels, IL-1β, IL-6, TNF-α cytokine expressions, and osteoclastogenesis. The results show that treatment with 78c on murine BMMs dose-dependently reduced CD38, reversed the decline of NAD^+^, and inhibited IL-1β, IL-6, and TNF-α pro-inflammatory cytokine levels induced by oral pathogen *Porphyromonas gingivalis* (*Pg*) or *Aggregatibacter actinomycetemcomitans* (*Aa*) or by advanced glycation end products (AGEs). Additionally, treatment with 78c dose-dependently suppressed osteoclastogenesis and bone resorption induced by RANKL. Treatment with 78c suppressed CD38, nuclear factor kappa-B (NF-κB), phosphoinositide 3-kinase (PI3K), and mitogen-activated protein kinases (MAPKs) induced by *Pg*, *Aa*, or AGEs, and suppressed podosome components (PI3K, Pyk2, Src, F-actin, integrins, paxillin, and talin) induced by RANKL. These results from our studies support the finding that the inhibition of CD38 by 78c is a promising therapeutic strategy to treat inflammatory bone loss diseases. However, treatment with a CD38 shRNA only significantly reduced IL-1β, IL-6, and TNF-α pro-inflammatory cytokine levels induced by AGEs. Compared with controls, it had limited effects on cytokine levels induced by *Pg* or *Aa*. Treatment with the CD38 shRNA enhanced RANKL-induced osteoclastogenesis, suggesting that 78c has some off-target effects.

## 1. Introduction

Cluster of Differentiation 38 (CD38) is a type II transmembrane ecto-enzyme ubiquitously expressed in most tissues and cells in mice and humans [1]. Predominantly, CD38 is highly expressed in B cells, plasma cells, natural killer cells, dendritic cells, T cells, monocytes, macrophages, neutrophils, and hematopoietic stem cells [1]. M1 macrophages express higher levels of CD38 than M2 macrophages [2]. CD38 is a nicotinamide adenine dinucleotide (NAD^+^) glycohydrolase, which breaks down NAD^+^ and generates nicotinamide (NAM), ADP-ribose (ADPR), and cyclic ADP-ribose (cADPR) [1,3,4,5]. Both ADPR and c-ADPR act as second messengers affecting Ca^2+^ mobilization [5,6]. NAD^+^ can be reduced to NADH via dehydrogenases, and NAD^+^ can be phosphorylated to NADP^+^ via NAD^+^ kinases [7]. The NAD^+^/NADH couple regulates cellular energy metabolism, glycolysis, and mitochondrial oxidative phosphorylation. In contrast, NADP^+^ and its reduced form NADPH are involved in maintaining redox homeostasis and supporting the biosynthesis of fatty and nucleic acids [7]. Previous studies [1,8] demonstrated that CD38 was robustly induced during inflammation. Depleting NAD^+^ is associated with many human diseases and pathological conditions, including diabetes, inflammation, aging, rheumatoid arthritis, and neurodegenerative disorders [1,9,10,11,12]. Therefore, CD38 has become a therapeutic target for treating infection, autoimmune diseases, aging, cancer, diabetes, and neurodegenerative disorders [1,3,4,5,13].

Inflammatory bone loss diseases, including rheumatoid arthritis, systemic lupus erythematosus, axial spondyloarthritis, psoriatic arthritis, and periodontitis, affect human beings. One of the characteristics of inflammatory bone loss diseases is that these diseases have enhanced pro-inflammatory cytokine (such as IL-1β, IL-6, and ΤNF-α) expressions. Patients with type II diabetes mellitus (T2DM) increase comorbidity with other inflammatory diseases, including periodontitis [14,15]. The comorbidity is associated with high levels of advanced glycation end products (AGEs) in patients with T2DM [16,17,18], which can further stimulate pro-inflammatory cytokine release induced by oral bacterial pathogens. *Porphyromonas gingivalis* (*Pg*) is a major oral pathogen in the initiation and development of severe forms of chronic periodontal disease with the ability to modulate various cytokines and NADPH-oxidase complex proteins [19]. *Pg* is found to be highly metabolically active in patients with periodontitis and can replicate and survive in the oral mucosa and invade deep tissues surrounding the bone [20,21]. *Aggregatibacter actinomycetemcomitans* (*Aa*) is another major oral pathogen associated with 90% localized aggressive periodontitis and from 30% to 50% of severe adult periodontitis affecting both juveniles and adults [22]. Microbial products and AGEs can activate toll-like receptors (TLRs) and their downstream signaling pathways [23,24,25,26,27], including NF-κB, PI3K, and MAPKs [including extracellular signal-regulated kinases (ERKs), c-Jun N-terminal kinase (JNK), and p38 MAPK], leading to the production of pro-inflammatory cytokines. 

Another significant characteristic of inflammatory bone loss diseases is that these diseases display enhanced osteoclastogenesis and bone loss response. Osteoclastogenesis is induced by cell adhesion and mononucleated monocytes (osteoclast precursors) fusion into multinucleated osteoclasts [28]. The receptor activator of NF-κB ligand (RANKL), the major osteoclast differentiation factor [29], binds with its receptor RANK, subsequently activating several osteoclastogenic factors, including the nuclear factor of activated T cells cytoplasmic calcineurin-dependent 1 (Nfatc1), cathepsin K (Ctsk), acid phosphatase 5 (Acp5), osteoclast stimulatory transmembrane protein (Ocstamp), and dendritic cell-specific transmembrane protein (Dcstamp) [30,31,32,33]. Activation of these osteoclastogenic factors promotes cell adhesion and fusion to form osteoclasts, leading to bone resorption. Podosomes, the primary cell adhesion units, are essential for cellular adhesion and fusion induced by RANKL [34,35]. Podosomes are composed of a filamentous actin (F-actin) core surrounded by a ring structure containing protein kinases (PI3K, Pyk2, Src) and adhesion proteins (including integrins, paxillin, and talin) [29,34,36]. Activating podosome protein kinases and adhesion proteins increases cellular adhesion and fusion to form osteoclasts, subsequently enhancing bone resorption [29,34,35,36]. 

Although it is well-known that CD38 is highly expressed in monocytes and macrophages, it is unclear if CD38 regulates pro-inflammatory cytokine release induced by oral bacterial pathogens or by AGEs. It is unclear if CD38 regulates osteoclastogenesis induced by RANKL. In this study, we determined the effects of a CD38-specific inhibitor (78c) or a CD38-specific shRNA on pro-inflammatory cytokine (IL-1β, IL-6, and ΤNF-α) levels induced by oral pathogens (*Pg* or *Aa*) or by AGEs in murine bone marrow-derived monocytes and macrophages (BMMs) isolated from male TALLYHO/JngJ mice [37,38]. Additionally, we determined the effects of a CD38-specific inhibitor (78c) or a CD38-specific shRNA on osteoclastogenesis and bone resorption induced by RANKL using murine BMMs derived from male TALLYHO/JngJ mice. 

## 2. Materials and Methods

### 2.1. Animals and Reagents

Six-week-old male and female TALLYHO/JngJ mice were purchased from Jackson Laboratory (Bar Harbor, ME, USA) and were bred at the Medical University of South Carolina. TALLYHO/JngJ mouse is an inbred polygenic model for T2DM, which mimics human T2DM maturity-onset development of hyperglycemia in contrast to juvenile-onset diabetes in monogenic diabetic animal models [37,38]). Although both male and female TALLYHO/JngJ mice showed hyperinsulinemia, only male TALLYHO/JngJ mice exhibited hyperglycemia and impaired glucose tolerance starting at 8 weeks old, while the female TALLYHO/JngJ mice showed normal glucose levels and did not display glucose intolerance, compared with C57BL/6J mice [37]. Therefore, only male TALLYHO/JngJ mice were used as a polygenic T2DM animal model [37,38]). TALLYHO/JngJ mice were fed with Lab Diet 5k52 and housed with a 12 h light/12 h dark cycle. This animal study was performed in accordance with ARRIVE guidelines for animal research and was approved by the Institutional Animal Care and Use Committee (IACUC) at the Medical University of South Carolina (IACUC-2021-01287). 78c was purchased from Tocris Bioscience (Minneapolis, MN, USA), dissolved in dimethyl sulfoxide (DMSO) as a 10 mM stock solution, and stored at −20 °C. An equal volume of DMSO (as compared to 10 mM 78c) was diluted in cell culture media and served as a vehicle control. The advanced glycation end product-BSA 10 mg/mL in sterilized filtered PBS was purchased from Millipore Sigma (St. Louis, MO, USA). 

### 2.2. Generation of L929 Conditioned Media

L929 (mouse fibroblast cell line) was purchased from the American Type Cell Culture Collection (ATCC, Manassas, VA, USA). L929 cells (5 × 10^5^) were plated in a T75 flask and cultured for six days in 30 mL of Dulbeco’s Modified Eagle Medium (DMEM, ThermoFisher Scientific, Waltham, MA, USA) supplemented with 10% fetal bovine serum (FBS), 100 U/mL penicillin, and 100 µg/mL streptomycin. The cell culture media was filtered through a 0.22 μM filter, aliquoted, and stored at −80 °C as L929 conditioned media (containing macrophage colony-stimulating factor, M-CSF). 

### 2.3. Generation of Bone Marrow-Derived Monocytes and Macrophages (BMMs)

Murine bone marrow cells were harvested from 8- to 12-week-old male TALLYHO/JngJ mice by flushing bone marrow cells from the tibia and femur using 10 mL cell culture media with a 10 mL syringe and 27 gauge needle (Becton Dickinson, Franklin Lakes, NJ, USA). The cell culture media is complete minimal essential media (MEM)-α (ThermoFisher Scientific) supplemented with 10% FBS, 100 U/mL penicillin and 100 µg/mL streptomycin. To remove tissue debris, the bone marrow cells were filtered through a 40 μM nylon cell strainer (ThermoFisher Scientific). Then, murine bone marrow cells were cultured in a complete MEM-α media supplement with 20% L929 conditioned media for three days. The attached bone marrow-stromal cells were discarded. The suspended bone marrow cells were transferred to new cell culture plates and cultured in complete MEM-α media supplement with 20% L929 conditioned media for another seven days until cells were differentiated into attached BMMs. Previous studies [39,40] showed that the adherent bone marrow-derived macrophages were usually better than 90% pure using macrophage markers such as CD11b and F4/80. One day before infection or stimulation, the cell culture media was changed to MEM-α media with 1% FBS without antibiotics. 

### 2.4. Bacterial Culture

Oral bacterial pathogens *Aggregatibacter actinomycetemcomitans* (*Aa*, ATCC 43718) and *Porphyromonas gingivalis* (*Pg*, ATCC 33277) were originally obtained the American Type Culture Collection. *Aa* was cultured in brain–heart infusion broth (Fisher Scientific, Suwanee, GA, USA) at 37 °C with 10% CO_2_. *Pg* was cultured to the early exponential phase in tryptic soy broth (Becton Dickinson, Sparks, MD, USA) supplemented with yeast extract (Becton Dickinson, 1 mg/mL), menadione (Chem-Implex Int’l Inc., Wood Dale, IL, USA, 1μg/mL), and hemin (Millipore Sigma, St. Louis, MO, USA, 5 μg/mL) at 37 °C under anaerobic conditions and harvested as previously described [19,41,42]. Briefly, the cell pellets of *Pg* or *Aa* were washed and resuspended with PBS before infection. *Pg* concentration was determined using a Klett-Summerson photometer (Bel-Art, Wayne, NJ, USA), followed by serial dilution and plating on tryptic soy agar plates supplemented with yeast extract (Becton Dickinson, 5 mg/mL), menadione (Chem-Implex Int’l Inc., Wood Dale, IL, USA, 1 μg/mL), hemin (Millipore Sigma, St. Louis, MO, USA, 5 μg/mL), and sheep blood (Hemostat Laboratories, Dixon, CA, USA) at 37 °C under anaerobic conditions. The K value 1.0 was equal to about 1.0 × 10^9^ CFU/mL of *Pg*. *Aa* bacterial concentration was determined by measuring bacterial optical density at 600 nm followed by serial dilution and plating on brain heart infusion agar plates (Fisher Scientific). OD_600_ = 1 was equal to about 3 × 10^8^ CFU/mL of *Aa*. Agar plate counts (CFU/mL) were used for both bacteria to calculate the multiplicity of infection (MOI), and MOI 20 was used for *Pg* or *Aa* infection of murine BMMs to detect noticeable cytokine expressions in the BMMs. A control group of cells were not infected with bacteria. 

### 2.5. Generation of shRNA Lentivirus

Murine CD38 shRNA plasmid DNA (TRCN0000068232, Millipore Sigma, Burlington, MA, USA), a control shRNA plasmid DNA (SHC002, Millipore Sigma), and Human Embryonic Kidney (HEK) 293 cells were obtained from the shRNA Shared Technology Resource at the Medical University of South Carolina. To generate lentiviral shRNA vectors, HEK293 cells were co-transfected with CD38 shRNA plasmid DNA or control shRNA plasmid DNA along with lentiviral packaging plasmids pCMV-VSV-G (Addgene, Cambridge, MA, USA) and pCMV-dR8.2 dvpr (Addgene) using a lipofectamine 2000 transfection reagent (2.9 μL/μg DNA, ThermoFisher Scientific). The ratio for the CD38 or control shRNA: pCMV-dR8.2 dvpr: pCMV-VSV-G was 15:7.5:15 for one 150 mm cell culture plate. The supernatant was collected after 72 h of transfection and ultracentrifuged at 25,000 rpm for 1.5 h at 4 °C using a Beckman ultracentrifuge (Beckman Coulter, Indianapolis, IN, USA). The viral pellet was resuspended in serum-free DMEM, and the viral titer was determined using a HIV-1 p24 Antigen ELISA kit (Zeptometrix, Buffalo, NY, USA). Purified lentiviral vectors were aliquoted and stored at −80 °C. Murine bone marrow cells were infected with a control or a CD38 shRNA lentiviral vector (MOI 10) for 72 h. The CD38 knockdown effect was evaluated using RT-qPCR or using Western blot, and was calibrated by either β-actin mRNA expression or pan-actin protein expression. 

### 2.6. NAD^+^ Assay

The NAD^+^ levels were determined using a NAD^+^/NADH cell-based assay kit according to the manufacturer’s instructions (Cayman Chemical, Ann Arbor, MI, USA). The NAD^+^ levels were calibrated by cell growth and viability determined using a CellTiter 96 Aqueous One Solution Cell Proliferation Assay (Promega, Madison, WI, USA).

### 2.7. Enzyme-Linked Immunosorbent Assay (ELISA)

IL-1β levels in cell lysates, IL-6, and TNF-α protein levels in cell culture media of BMMs were quantified using ELISA kits (R&D Systems, Minneapolis, MN, USA). The concentration of cytokines was normalized by protein concentration, which was determined using a DC protein Assay Kit (Bio-Rad Laboratories, Hercules, CA, USA) in cell lysates (100 μL RIPA cell lysis buffer (Cell Signaling Technology, Danvers, MA, USA)/well in a 12-well cell culture plate). 

### 2.8. Osteoclastogenesis Assay and Bone Resorption Assay

As described above, murine bone marrow cells were harvested from 8- to 12-week-old male TALLYHO/JngJ mice. Bone marrow cells were then cultured in a complete MEM-α media supplement with 20% L929 conditioned media for two days. The suspended bone marrow cells were plated in a new 96-well cell culture plate (for osteoclastogenesis assay) or a calcium phosphate-coated 48-well plate (for bone resorption assay, Cosmo Bio USA, Carlsbad, CA, USA). Cells were treated with either vehicle (diluted DMSO), 78c, a control shRNA, or a CD38 shRNA. They were cultured in complete MEM-α media containing 20% conditioned L929 media and recombinant murine RANKL (1000 ng/mL, PeproTech, Cranbury, NJ, USA). A control group of cells were cultured in complete MEM-α media containing only 20% conditioned L929 media. The media was changed every other day. After RANKL treatment for five days, osteoclasts were stained using Tartrate-Resistant Acid Phosphatase (TRAP) staining using a leukocyte acid phosphatase kit (Millipore Sigma). Pictures were taken using a VWR inverted microscope. Image analysis was performed using Adobe Photoshop CS5 Extended Version 12.1 software (Adobe, San Jose, CA, USA). For the bone resorption assay, eight days after RANKL treatment, cells were removed by treatment with 5% sodium hypochlorite for 5 min. After washing and drying the plate, osteoclast resorption pit images were taken using a VWR inverted microscope and analyzed using Adobe Photoshop CS5 Extended Version 12.1 software. 

### 2.9. RNA Extraction and Real-Time PCR

According to the manufacturer’s instructions, total RNA was isolated from murine bone marrow cells using TRIZOL (ThermoFisher Scientific). Complementary DNA was synthesized using a TaqMan reverse transcription kit (Life Technologies, Carlsbad, CA, USA) using the total RNA (1 μg). Real-time PCR was performed using a StepOnePlus Real-Time PCR System (Life Technologies). PCR conditions used were as follows: 50 °C for 2 min, 95 °C for 10 min, and 40 cycles of 95 °C for 15 s, and 60 °C for 1 min. The following amplicon primers were obtained from Life Technologies: CD38 (Mm00483143_m1), Nfatc1 (Mm00479445_m1), Ctsk (Mm00484039_m1), Acp5 (Mm00475698_m1), Oscar (Mm00558665_m1), Ocstamp (Mm00512445_m1), Dcstamp (Mm04209236_m1), and β-actin (Mm02619580_g1). Amplicon concentration was determined using threshold cycle values compared with standard curves for each primer. Sample mRNA levels were normalized to an endogenous control β-actin expression and were expressed as fold changes compared with control groups. 

### 2.10. Western Blot Analysis

Per the manufacturer’s guidance, protein was extracted from murine BMMs using a RIPA cell lysis buffer (Cell Signaling Technology). Total protein (25 μg) was loaded on 10% Tris-HCl gels, electro-transferred to nitrocellulose membranes, blocked, and incubated overnight at 4 °C with primary antibodies. The antibodies to p-PI3K, p-ERK, p-JNK, p-p38, p-NF-κB p65, p-Src, p-Pyk2, integrin β3, and pan-actin were purchased from Cell Signaling Technology (Danvers, MA, USA). An antibody to F-actin was obtained from Abcam (Cambridge, MA, USA). An antibody to paxillin was purchased from Santa Cruz Biotechnology Inc. (Dallas, TX, USA). All primary antibodies were diluted in ratios of 1:500 or 1:1000. After washing, the nitrocellulose membranes were incubated at room temperature for one hour with horseradish peroxidase-conjugated secondary antibodies (Cell Signaling Technology) and developed using SuperSignal West Pico Chemiluminescent Substrate (ThermoFisher Scientific). Digital images and protein densitometry were analyzed with a G-BOX chemiluminescence imaging system (Syngene, Frederick, MD, USA). 

### 2.11. Statistical Analysis

All experiments were performed in triplicate with murine bone marrow cells. Data were checked for normality using a QQ plot. If the data were normally distributed, the data were analyzed either using a one-way ANOVA with Dunnett’s or Tukey’s multiple comparisons tests or by using a two-way ANOVA with multiple comparisons if comparing more than three data groups. The data were analyzed using an unpaired *t*-test with Welch’s correction if two data groups were compared. If the data were not normally distributed, the data were analyzed using a non-parametric ANOVA with the Kruskal–Wallis correction. All statistical tests were performed using GraphPad Prism software (Version 10.4.0, GraphPad Software Inc., La Jolla, CA, USA). Values are expressed as means ± standard error of the means (SEM) of three independent experiments. A *p*-value of 0.05 or less was considered significant. 

## 3. Results

### 3.1. Inhibition of CD38 by 78c Reduced CD38, Reversed the Decline of NAD^+^, and Suppressed IL-1β, IL-6, and TNF-α Proinflammatory Cytokines in Macrophages Infected by Oral Pathogens or Stimulated by AGEs

**Figure 1:**
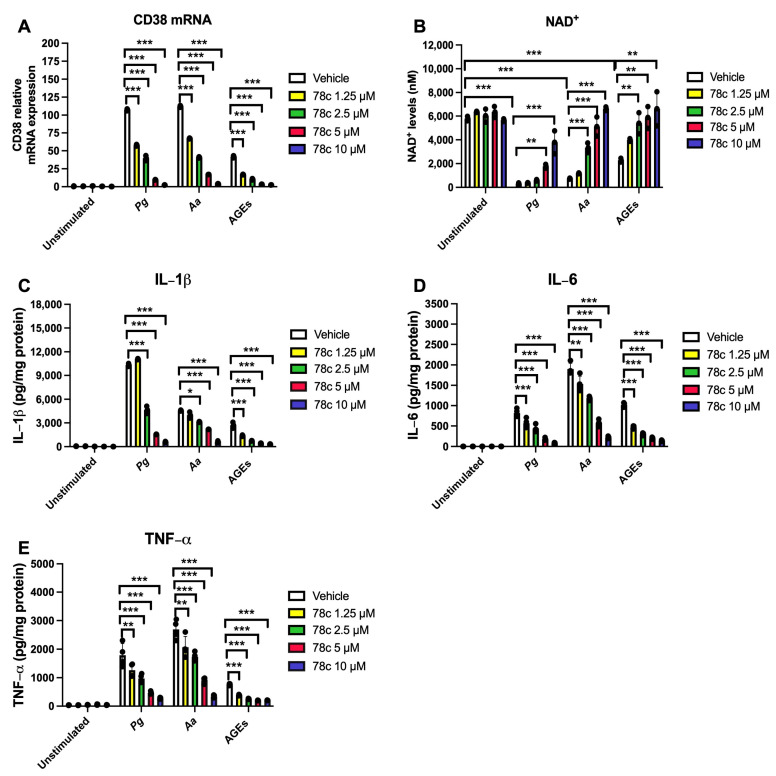
Inhibition of CD38 by 78c reduced CD38 gene expression, reversed the decline of NAD^+^, and suppressed IL-1β, IL-6, and TNF-α pro-inflammatory cytokines in murine BMMs infected by oral pathogens or stimulated by AGEs. Murine BMMs were treated with vehicles (diluted DMSO) or 78c (1.25 to 10 μM) with or without infection with *Porphyromonas gingivalis* (*Pg*), *Aggregatibacter actinomycetemcomitans* (*Aa*), or stimulation by AGEs for 24 h. (**A**) The mRNA levels of CD38 were evaluated using RT-q-PCR and normalized by β-actin expression. (**B**) NAD^+^ levels were measured and calibrated by cell growth and viability. (**C**) IL-1β, (**D**) IL-6, and (**E**) TNF-α levels were measured using ELISA and calibrated by protein concentration in cell lysate. Statistics were analyzed using a two-way ANOVA with Dunnett’s multiple comparisons test (*n* = 3, * *p* < 0.05, ** *p* < 0.01, *** *p* < 0.001).

To determine the effects of the CD38 inhibitor (78c) on the levels of CD38, NAD^+^, and pro-inflammatory cytokine levels in murine BMMs with or without infection by oral pathogen *Porphyromonas gingivalis* (*Pg*) or *Aggregatibacter actinomycetemcomitans* (*Aa*) or stimulation by AGEs, murine BMMs were treated with vehicle (diluted DMSO) or 78c (1.25 μM to 10 μM), and were either unstimulated, infected with oral bacterial pathogen *Pg* or *Aa* (MOI 20), or stimulated with AGEs (10 μg/mL) for 24 h. As shown in Figure 1A, in cells treated with vehicle and either infected with *Pg*, *Aa*, or stimulated with AGEs, CD38 mRNA levels increased to an average of 107-fold, 112-fold, and 42-fold, respectively, compared with cells treated with only vehicle. Inhibition of CD38 by 78c dose-dependently reduced CD38 mRNA levels in cells with bacterial infection or AGEs stimulation. 78c (1.25 μM, 2.5 μM, 5 μM, and 10 μM) reduced CD38 mRNA levels by 46.3%, 62.9%, 91.0%, and 97.8% induced by *Pg*, decreased CD38 mRNA levels by 40.0%, 63.6%, 84.8%, and 96.3% induced by *Aa*, and attenuated CD38 mRNA levels by 59.3%, 75.1%, 91.8%, and 93.9% induced by AGEs, respectively, compared with controls. Additionally, in cells treated with a vehicle and either infected with *Pg* or *Aa* or stimulated with AGEs, the NAD^+^ levels significantly decreased compared with cells treated with a vehicle only (Figure 1B, *** *p* < 0.001). Treatment with 78c dose-dependently prevented the decline of NAD^+^ levels in cells infected with oral pathogen *Pg* or *Aa* or stimulated by AGEs (Figure 1B). Furthermore, treatment with 78c dose-dependently suppressed IL-1β, IL-6, and TNF-α pro-inflammatory cytokine levels in murine BMMs infected with *Pg* or *Aa* or stimulated with AGEs (Figure 1C–E). These results support that the inhibition of CD38 by 78 dose-dependently suppressed CD38, reversed the decline of NAD^+^, and reduced IL-1β, IL-6, and TNF-α pro-inflammatory cytokine levels in murine BMMs infected with oral pathogens or stimulated with AGEs.

### 3.2. Inhibition of CD38 by 78c Reduced the Expressions of NF-kB, PI3K, MAPK, and CD38 Induced by Oral Pathogens or AGEs in Murine BMMs

**Figure 2:**
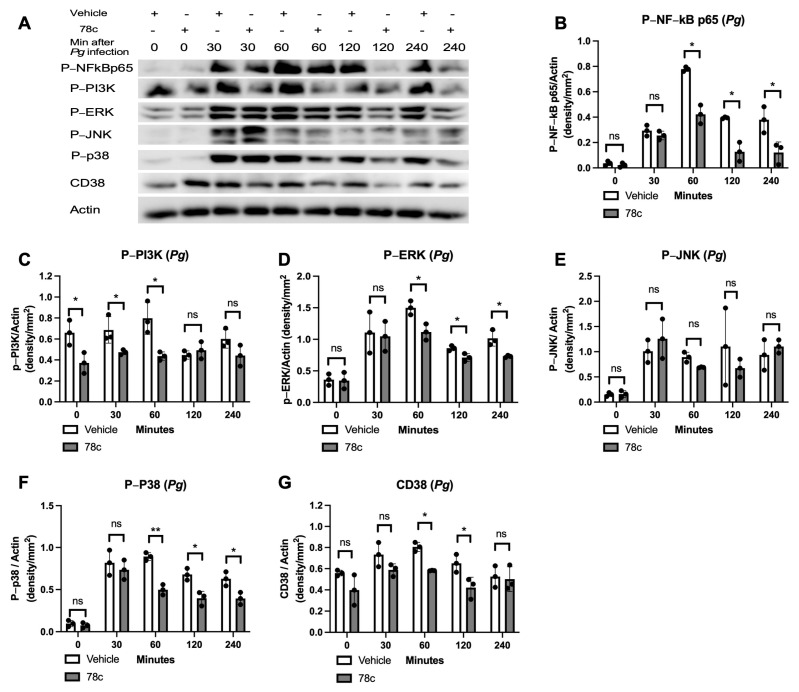
Inhibition of CD38 by 78c reduced NF-kB, PI3K, ERK, p38 MAPK, and CD38 protein expressions induced by the oral pathogen *Porphyromonas gingivalis* (*Pg*). Murine BMMs were treated with vehicle (diluted DMSO) or 78c (10 μM) with or without infection with *Pg* from 30 min to 240 min. (**A**) Protein levels of p-NFκBp65, p-PI3K, p-ERK, p-JNK, p-p38, CD38, and pan-actin in cell lysate were determined using Western blot. Protein densitometry of p-NFκBp65 (**B**), p-PI3K (**C**), p-ERK (**D**), p-JNK (**E**), p-p38 (**F**), and CD38 (**G**) were evaluated. Statistics were assessed using an unpaired *t*-test to compare vehicle vs. 78c treatment using Welch’s correction (*n* = 3, ns: no significance, * *p* < 0.05, ** *p* < 0.01).

**Figure 3:**
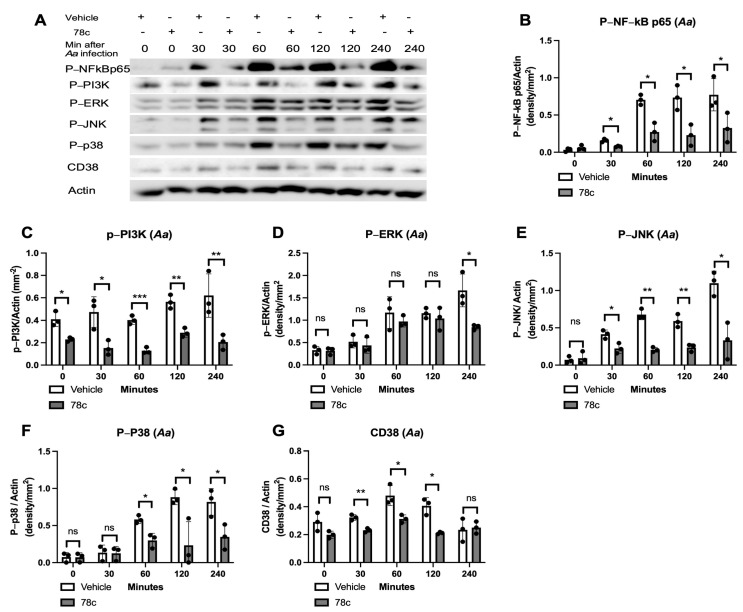
Inhibition of CD38 by 78c reduced NF-kB, PI3K, ERK, JNK, p38 MAPK, and CD38 protein expressions induced by oral pathogen *Aggregatibacter actinomycetemcomitans* (*Aa*). Murine BMMs were treated with vehicle (diluted DMSO) or 78c (10 μM) with or without infection with *Aa* from 30 min to 240 min. (**A**) Protein levels of p-NFκBp65, p-PI3K, p-ERK, p-JNK, p-p38, CD38, and pan-actin in cell lysate were determined using Western blot. Protein densitometry of p-NFκBp65 (**B**), p-PI3K (**C**), p-ERK (**D**), p-JNK (**E**), p-p38 (**F**), and CD38 (**G**) were evaluated. Statistics were assessed using an unpaired *t*-test with Welch’s correction (*n* = 3, ns: no significance, * *p* < 0.05, ** *p* < 0.01, *** *p* < 0.001).

**Figure 4:**
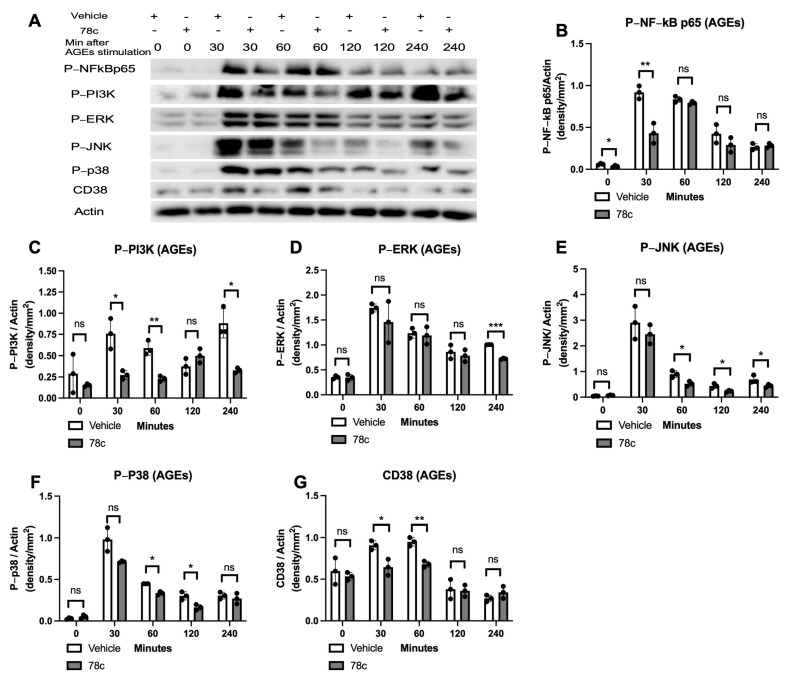
Inhibition of CD38 by 78c reduced NF-kB, PI3K, ERK, JNK, p38 MAPK, and CD38 protein expressions induced by advanced glycation end products (AGEs). Murine BMMs were treated with vehicle (diluted DMSO) or 78c (10 μM) with or without stimulation by AGEs (10 μg/mL) from 30 min to 240 min. (**A**) Protein levels of p-NFκBp65, p-PI3K, p-ERK, p-JNK, p-p38, CD38, and pan-actin in cell lysate were determined using Western blot. Protein densitometry of p-NFκBp65 (**B**), p-PI3K (**C**), p-ERK (**D**), p-JNK (**E**), p-p38 (**F**), and CD38 (**G**) were evaluated. Statistics were assessed using an unpaired *t*-test with Welch’s correction (*n* = 3, ns: no significance, * *p* < 0.05, ** *p* < 0.01, *** *p* < 0.001).

To determine how 78c alleviated pro-inflammatory cytokine release induced by oral pathogens or AGEs, murine BMMs were treated with vehicle or 78c (10 μM). They were either uninfected, infected with *Pg* or *Aa*, or stimulated by AGEs from 30 to 240 min. The protein levels of p-NF-κBp65, p-PI3K, p-ERK, p-JNK, p-p38 MAPK, CD38, and control pan-actin were quantified using Western blot in cell lysates. In cells infected with *Pg*, treatment with 78c significantly reduced p-NFκBp65 at 60, 120, and 240 min after *Pg* infection (Figure 2A,B, * *p* < 0.05), significantly decreased p-PI3K in cells either without *Pg* infection or infected with *Pg* for 30 or 60 min (Figure 2A,C, * *p* < 0.05), significantly reduced p-ERK at 60, 120, and 240 min after *Pg* infection (Figure 2A,D, * *p* < 0.05), significantly decreased p-p38 MAPK at 60, 120, and 240 min after *Pg* infection (Figure 2A,F, * *p* < 0.05, ** *p* < 0.01), and considerably reduced CD38 protein levels at 60 and 120 min after *Pg* infection (Figure 2A,G, * *p* < 0.05). However, there were no significant differences in p-JNK expressions in cells with or without *Pg* infection (Figure 2A,E). In cells infected with *Aa*, treatment with 78c significantly reduced p-NFκBp65 at 30, 60, 120, and 240 min after *Aa* infection (Figure 3A,B, * *p* < 0.05), significantly decreased p-PI3K in cells without infection or that were infected with *Aa* for 30, 60, 120, and 240 min (Figure 3A,C, * *p* < 0.05, ** *p* < 0.01, *** *p* < 0.001), significantly decreased p-ERK at 240 min after *Aa* infection (Figure 3A,D, * *p* < 0.05), significantly reduced p-JNK at 30, 60, 120, and 240 min after *Aa* infection (Figure 3A,E, * *p* < 0.05, ** *p* < 0.01), significantly decreased p-p38 MAPK at 60, 120, and 240 min after *Aa* infection (Figure 3A,F, * *p* < 0.05), and significantly decreased CD38 at 30, 60, and 120 min after *Aa* infection (Figure 3A,G, * *p* < 0.05, ** *p* < 0.01). In cells stimulated with AGEs, treatment with 78c significantly reduced p-NFκBp65 in cells without stimulation or stimulated with AGEs for 30 min (Figure 4A,B, * *p* < 0.05, ** *p* < 0.01), significantly decreased p-PI3K in cells stimulated with AGEs for at 30, 60, and 240 min (Figure 4A,C, * *p* < 0.05, ** *p* < 0.01), significantly decreased p-ERK at 240 min after AGEs stimulation (Figure 4A,D, *** *p* < 0.001), significantly reduced p-JNK in cells stimulated with AGEs for 60, 120, and 240 min (Figure 4A,E, * *p* < 0.05), significantly attenuated p-p38 MAPK at 60 min and 120 min after AGEs stimulation (Figure 4A,F, * *p* < 0.05), and significantly decreased CD38 at 30 and 60 min after AGEs stimulation (Figure 4A,G, * *p* < 0.05, ** *p* < 0.01). These results demonstrate that treatment with 78c suppressed NF-kB, PI3K, and MAPK signaling pathways induced by oral pathogens or AGEs.

### 3.3. Inhibition of CD38 by 78c Suppressed Osteoclastogenesis and Bone Resorption Induced by RANKL

**Figure 5:**
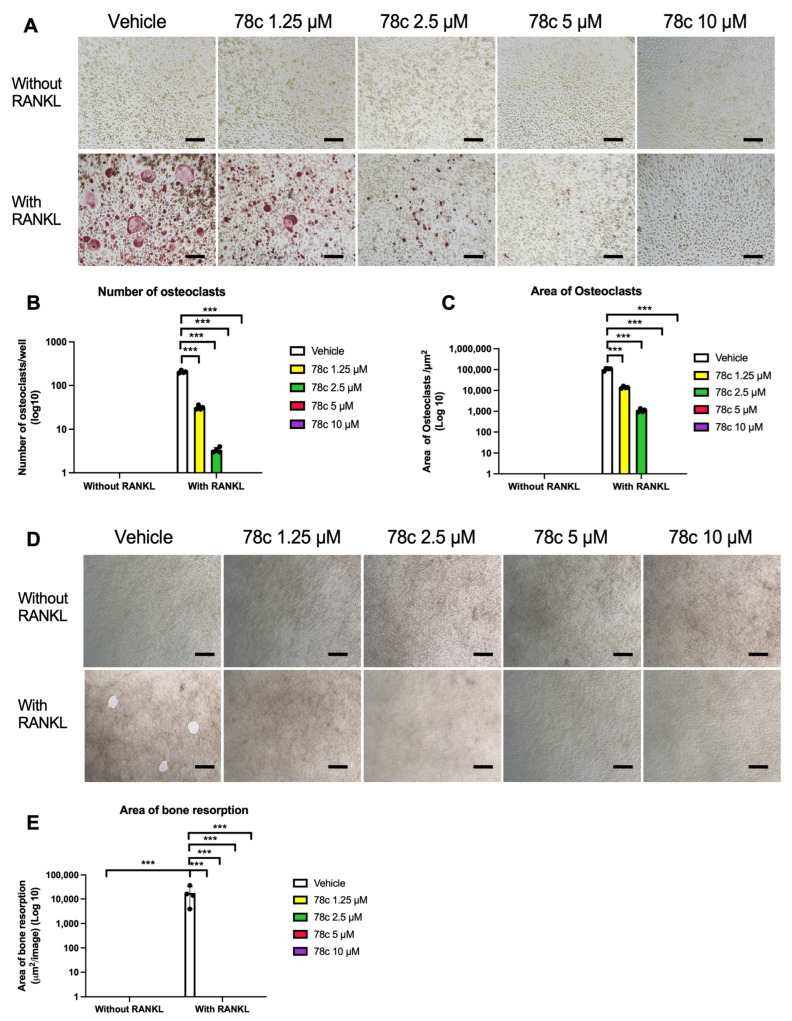
Treatment with 78c suppressed osteoclastogenesis and bone resorption induced by RANKL. Murine bone marrow cells were treated with vehicle (diluted DMSO) or 78c (1.25 to 10 μM) with or without stimulation by RANKL for five days (in a 96-well cell culture plate for osteoclastogenesis assay) or for eight days (in a calcium phosphate-coated 48-well plate for bone resorption assay). (**A**) Representative images show TRAP-stained cells at the 100× magnification view. Scale bars represent 50 μM. (**B**) Number of TRAP^+^ multinucleated (more than three nuclei) osteoclasts/well (96-well). (**C**) Total areas of osteoclasts/image were quantified. (**D**) Representative images show osteoclast resorption pits at 200× magnification view. Scale bars represent 100 μM. (**E**) Total areas of osteoclast resorption pits/images were quantified. Statistics were analyzed using a non-parametric ANOVA with the Kruskal–Wallis correction (*n* = 4, *** *p* < 0.001).

To determine the effect of 78c on osteoclastogenesis and bone resorption, murine BMMs were treated with either vehicle (diluted DMSO) or 78c (from 1.25 μM to 10 μM) and cultured for five days (for osteoclastogenesis assay) or eight days (for bone resorption assay) in MEM-α media containing 20% conditioned L929 media with or without RANKL (1000 ng/mL). As shown in Figure 5, tartrate-resistant acid phosphatase (TRAP) staining of osteoclasts revealed that treatment with 78c dose-dependently suppressed osteoclastogenesis induced by RANKL. There were only a few TRAP-stained multinucleated osteoclasts in cells treated with 1.25 μM 78c with RANKL stimulation and only some TRAP-stained single-nucleated cells displayed in cells treated with 2.5 μM 78c with RANKL stimulation. Treatment with 5 μM or 10 μM 78c further suppressed TRAP staining in murine BMMs. Treatment with 78c (1.25 μM to 10 μM) significantly suppressed the number of osteoclasts and the area of osteoclasts in cells with RANKL stimulation (Figure 5B,C, *** *p* < 0.001). Additionally, treatment with 78c (1.25 μM to 10 μM) completely suppressed bone resorption induced by RANKL (Figure 5D). Treatment with 78c significantly reduced the area of bone resorption pits induced by RANKL (Figure 5E, *** *p* < 0.001). 

### 3.4. Inhibition of CD38 by 78c Reduced the mRNA Levels of Osteoclastogenic Factors, Including Nfatc1, Ctsk, Acp5, Oscar, Ocstamp, and Dcstamp, Induced by RANKL

**Figure 6:**
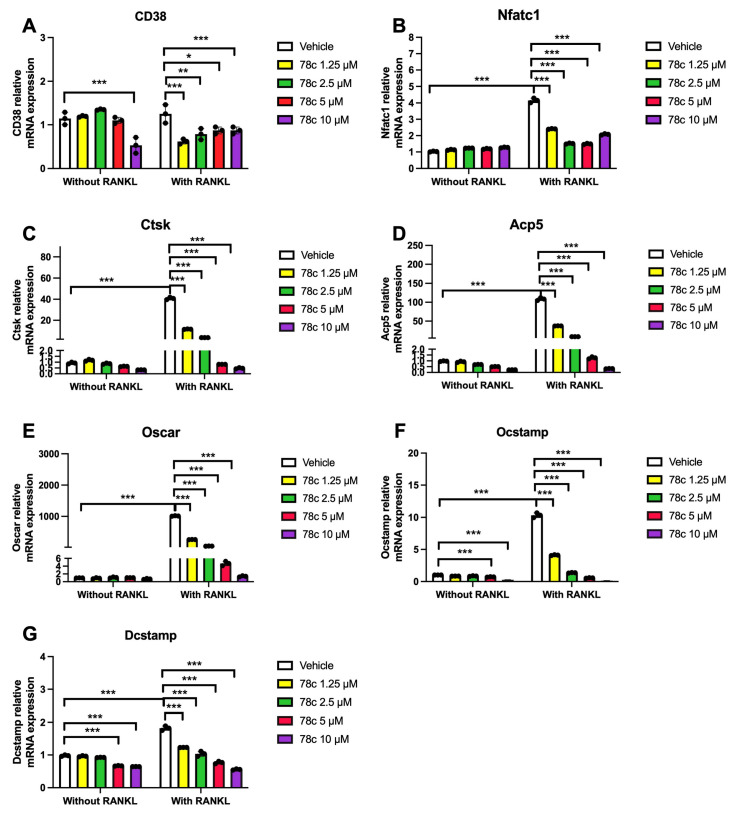
Inhibition of CD38 by 78c attenuated CD38, Nfatc1, Ctsk, Acp5, Oscar, Ocstamp, and Dcstamp mRNA expressions induced by RANKL. Murine bone marrow cells were treated with vehicle (diluted DMSO) or 78c (1.25 to 10 μM) with or without stimulation by RANKL for four days. (**A**) CD38 mRNA, (**B**) Nfatc1 mRNA, (**C**) Ctsk mRNA, (**D**) Acp5 mRNA, (**E**) Oscar mRNA, (**F**) Ocstamp mRNA, and (**G**) Dcstamp mRNA levels were quantified using RT-PCR and normalized by β-actin expression. Statistics were analyzed using an ordinary two-way ANOVA with multiple comparisons (*n* = 3, * *p* < 0.05, ** *p* < 0.01, *** *p* < 0.001).

To determine how 78c regulates osteoclastogenesis induced by RANKL, we quantified the mRNA levels of CD38 and osteoclastogenic factors, including *Nfatc1*, *Ctsk*, *Acp5*, *Oscar*, *Ocstamp*, and *Dcstamp* in murine BMMs with or without RANKL stimulation. Treatment with 78c (1.25 to 10 μM) significantly reduced CD38 mRNA expression in cells stimulated with RANKL (* *p* < 0.05, ** *p* < 0.01, *** *p* < 0.001), while only 10 μM significantly suppressed CD38 mRNA levels in cells without RANKL stimulation (Figure 6A, *** *p* < 0.001). In cells treated with vehicle, treatment with RANKL significantly enhanced *Nfatc1*, *Ctsk*, *Acp5*, *Oscar*, *Ocstamp*, and *Dcstamp* osteoclastogenic factors compared the mRNA levels in cells treated with vehicle only (Figure 6B–G, *** *p* < 0.001). Treatment with 78c dose-dependently reduced the mRNA levels of these osteoclastogenic factors induced by RANKL. Treatment with 1.25 μM, 2.5 μM, 5 μM, and 10 μM 78c decreased *Nfatc1* by 41.9%, 63.5%, 64.1%, and 50.0%, respectively (Figure 6B), decreased *Ctsk* by 71.5%, 91.3%, 98.0%, and 98.8%, respectively (Figure 6C), reduced *Acp5* by 65.5%, 91.9%, 98.8%, and 99.7%, respectively (Figure 6D), decreased *Oscar* by 74.2%, 94.6%, 99.5%, and 99.9%, respectively (Figure 6E), reduced *Ocstamp* by 60.3%, 86.9%, 94.6%, and 98.7%, respectively (Figure 6F), and decreased *Dcstamp* by 32.4%, 43.2%, 57.8%, and 69.5%, respectively (Figure 6G). These results support that 78c inhibited osteoclastogenesis induced by RANKL by suppressing osteoclastogenic factors, including *Nfatc1*, *Ctsk*, *Acp5*, *Oscar*, *Ocstamp*, and *Dcstamp* induced by RANKL.

### 3.5. Inhibition of CD38 by 78c Decreased Podosome-Associated Protein Kinase and Adhesion Protein Levels Induced by RANKL

**Figure 7:**
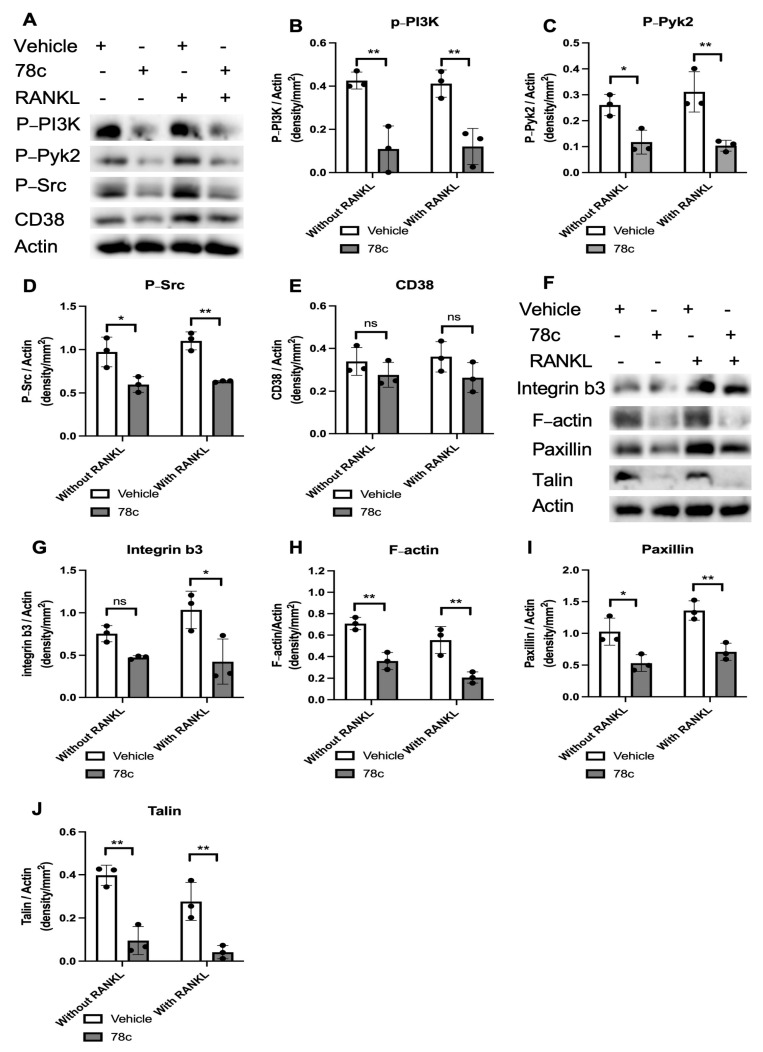
Inhibition of CD38 by 78c inhibited podosome components (PI3K, Pyk2, Src, integrin β3, F-actin, paxillin, and talin) induced by RANKL. Murine bone marrow cells were treated with vehicle (diluted DMSO) or 78c (5 μM) with or without RANKL stimulation for four days. (**A**) Protein levels of p-PI3K, p-Pyk2, p-Src, CD38, and pan-actin protein levels were evaluated using Western blot. Protein densitometry of (**B**) p-PI3K, (**C**) p-Pyk2, (**D**) p-Src, and (**E**) CD38 were evaluated. (**F**) Protein levels of integrin β3, F-actin, paxillin, talin, and pan-actin protein levels were evaluated using Western blot. Protein densitometry of integrin β3 (**G**), F-actin (**H**), paxillin (**I**), and talin (**J**) were evaluated. Statistics were evaluated using an unpaired *t*-test with Welch’s correction (*n* = 3, ns: no significance, * *p* < 0.05, ** *p* < 0.01).

Because the activation of podosome-associated protein kinases (PI3K, Pyk2, Src) and adhesion proteins (including integrins, F-actin, paxillin, and talin) are essential for cellular adhesion and fusion to form multinucleated osteoclasts, we hypothesize that treatment with 78c could inhibit podosome-associated protein kinases and adhesion protein levels induced by RANKL. As shown in Figure 7A–D, treatment with 78c reduced p-PI3K, p-Pyk2, and p-Src protein levels in murine BMMs with or without RANKL stimulation. Although there was a trend of reduction in CD38 protein in murine BMMs treated with 78c compared with vehicle treatment, no significance existed in CD38 protein levels between vehicle and 78c treatment in cells with or without RANKL stimulation. Accordingly, we observed reductions in integrin β3, F-actin, paxillin, and talin protein levels in murine bone marrow cells with or without RANKL stimulation (Figure 7F–J). These results support the notion that 78c inhibited osteoclastogenesis induced by RANKL by suppressing podosome-associated protein kinases (PI3K, Pyk2, Src) and adhesion protein (integrin β3, F-actin, paxillin, and talin) levels in murine BMMs.

### 3.6. 78c Displays Some Off-Target Effects in Inhibiting IL-1β, IL-6, and TNF-α Cytokine Levels Induced by Oral Pathogens

**Figure 8:**
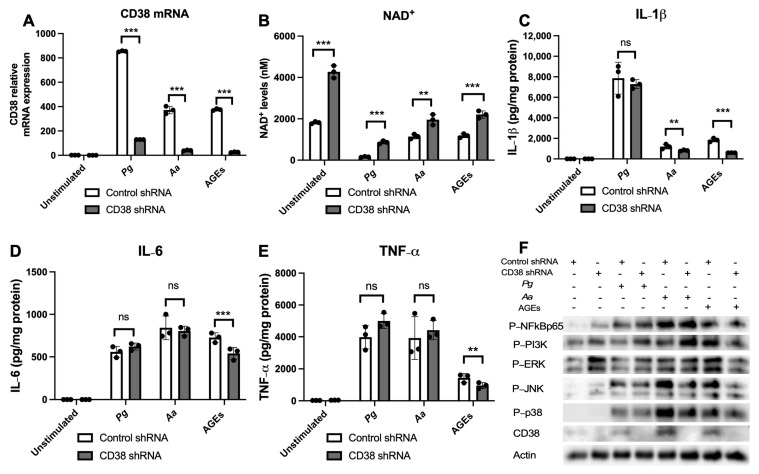
Effect of a CD38 shRNA on CD38 mRNA, NAD^+^, IL-1β, IL-6, and TNF-α cytokine levels, NF-κB, PI3K, MAPK, and CD38 Protein levels induced by oral pathogen *Porphyromonas gingivalis* (*Pg*), *Aggregatibacter actinomycetemcomitans* (*Aa*), or by advanced glycation end products (AGEs) compared with controls. Murine BMMs were treated with a control shRNA or a CD38 shRNA lentiviral vector (MOI 10). At 72 h after lentiviral infection, cells were untreated, infected with *Pg* or *Aa*, or stimulated by AGEs for 24 h (for CD38 mRNA assay, NAD^+^ assay, and IL-1β, IL-6, TNF-α cytokine assays) or 2 h (for Western blot assay). (**A**) CD38 mRNA levels were evaluated using RT-q-PCR and normalized by β-actin expression. (**B**) NAD^+^ levels were measured and calibrated by cell growth and viability. (**C**) IL-1β, (**D**) IL-6, and (**E**) TNF-α levels were measured using ELISA and calibrated by protein concentration in cell lysate. Statistics were analyzed using an unpaired *t*-test with Welch’s correction (*n* = 3, ns: no significance, ** *p* < 0.01, *** *p* < 0.001). (**F**) Protein levels of p-NFκBp65, p-PI3K, p-ERK, p-JNK, p-p38, CD38, and pan-actin in cell lysate were determined using Western blot.

To determine if 78c possesses some off-target effects in suppressing pro-inflammatory cytokine production induced by oral pathogens or AGEs, we treated murine BMMs with a control shRNA or a CD38 shRNA lentiviral vector. At 72 h after the lentiviral infection, murine BMMs were either unstimulated, infected with *Pg* or *Aa*, or stimulated by AGEs for 24 h. As shown in Figure 8A, treatment with the CD38 shRNA reduced the CD38 mRNA levels by 84.8%, 89.1%, and 93.1% induced by *Pg*, *Aa*, or AGEs, respectively. Treatment with the CD38 shRNA significantly enhanced NAD^+^ levels in murine BMMs either without stimulation, infected with *Pg* or *Aa*, or stimulated by AGEs (Figure 8B, ** *p* < 0.01, *** *p* < 0.001). However, treatment with the CD38 shRNA only significantly reduced IL-1β, IL-6, and TNF-α cytokine levels induced by AGEs and slightly decreased IL-1β levels induced by *Aa* (Figure 8C–E, ** *p* < 0.01, *** *p* < 0.001). *Pg* induced no significant difference in IL-1β levels between cells treated with the CD38 shRNA or the control shRNA (Figure 8C–E). No significant difference existed in either IL-6 or TNF-α levels induced by *Pg* or *Aa* between cells treated with the CD38 shRNA or the control shRNA (Figure 8C–E). Western blot imaging showed that in murine BMMs stimulated by AGEs, the p-NFκBp65 level was similar between the CD38 shRNA and the control shRNA treatment. Instead, we observed a reduction in p-PI3K, p-ERK, p-JNK, and p38 MAPK in cells treated with the CD38 shRNA compared with controls (Figure 8F). In contrast, in cells infected with *Pg* or *Aa*, the p-NFκBp65 level was similar between the CD38 shRNA and the control shRNA treatment. In contrast, we observed a slightly increased level of p-PI3K, a comparable level of p-ERK, and slightly reduced levels of p-JNK and p-p38 in murine BMMs treated with the CD38 shRNA compared with controls. These results support the notion that the CD38 inhibitor (78c) has some off-target effects in inhibiting IL-1β, IL-6, and TNF-α cytokine levels induced by the oral pathogen *Pg* or *Aa*.

### 3.7. 78c Has Some Off-Target Effects in Suppressing Osteoclastogenesis and Bone Resorption Induced by RANKL

**Figure 9:**
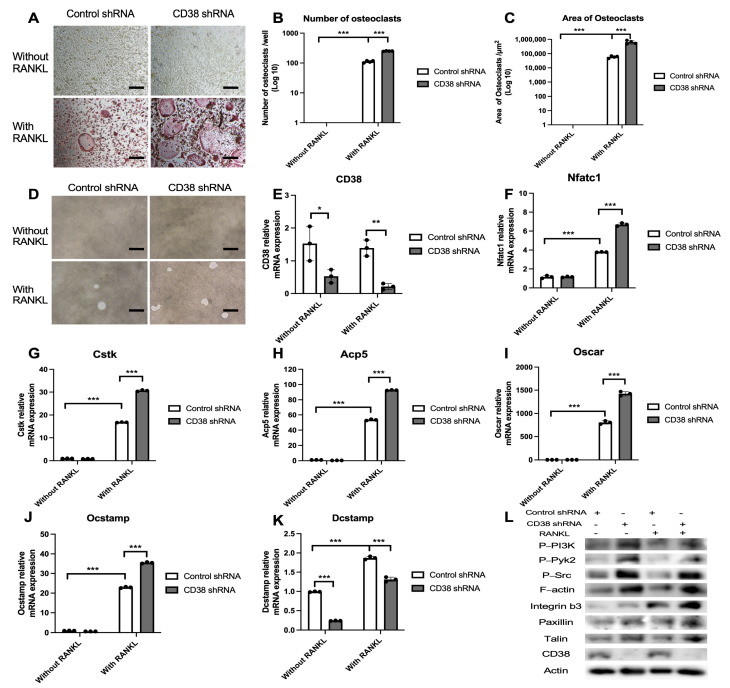
Effect of a CD38 shRNA on osteoclastogenesis and bone resorption induced by RANKL. Murine bone marrow cells were treated with a control shRNA or a CD38 shRNA lentiviral vector (MOI 10). At 24 h after lentiviral infection, the cells were unstimulated or stimulated with RANLK for five days (in a 96-well cell culture plate for osteoclastogenesis assay), eight days (in a calcium phosphate-coated 48-well plate for bone resorption assay), or four days (for RT-PCR assay and Western blot assay). (**A**) Representative images show TRAP-stained cells at the 100× magnification view. Scale bars represent 50 μM. (**B**) Number of TRAP^+^ multinucleated (more than three nuclei) osteoclasts/well (96-well) (*n* = 4). (**C**) Total areas of osteoclast/image were quantified (*n* = 4). (**D**) Representative images show osteoclast resorption pits at 200× magnification view. Scale bars represent 100 μM. (**E**) CD38 mRNA level, (**F**) Nfatc1 mRNA level, (**G**) Ctsk mRNA level, (**H**) Acp5 mRNA level, (**I**) Oscar mRNA level, (**J**) Ocstamp mRNA level, and (**K**) Dcstamp mRNA levels were quantified using RT-PCR and normalized by β-actin expression. Statistics were analyzed using an ordinary one-way ANOVA with Tukey’s multiple comparisons test (*n* = 3, * *p* < 0.05, ** *p* < 0.01, *** *p* < 0.001). (**L**) Protein levels of p-PI3K, p-Pyk2, p-Src, F-actin, integrin β3, paxillin, talin, CD38, and pan-actin protein levels were evaluated using Western blot.

To determine if 78c possesses some off-target effects in suppressing osteoclastogenesis and bone resorption induced by RANKL, we treated murine bone marrow cells with a control shRNA or a CD38 shRNA lentiviral vector. At 24 h after the lentiviral infection, cells were treated with or without RANKL (1000 ng/mL) for five days (for osteoclastogenesis assay) or eight days (for bone resorption assay). As shown in Figure 9A–C, treatment with the CD38 shRNA enhanced the number and the area of osteoclasts induced by RANKL compared with controls. Treatment with the CD38 shRNA also enhanced bone resorption induced by RANKL compared with controls (Figure 9D). RNA analysis revealed that the CD38 shRNA significantly reduced CD38 mRNA level in murine bone marrow cells with or without RANKL stimulation compared with controls (Figure 9E, * *p* < 0.05, ** *p* < 0.01). However, treatment with the CD38 shRNA significantly increased the mRNA levels of Nfatc1, Ctsk, Acp5, Oscar, and Ocstamp induced by RANKL compared with controls (Figure 9F–J, *** *p* < 0.001). In contrast, treatment with the CD38 shRNA significantly reduced Dcstamp mRNA levels in cells with or without RANKL stimulation (Figure 9K, *** *p* < 0.001). Western blot results showed that treatment with the CD38 shRNA increased podosome-associated protein kinases (PI3K, Pyk2, Src) and adhesion protein (integrin β3, F-actin, paxillin, and talin) levels in murine bone marrow cells with or without RANKL stimulation (Figure 9L). These results support that 78c possesses some off-target effects in suppressing osteoclastogenesis and bone resorption induced by RANKL compared with the CD38 shRNA treatment.

## 4. Discussion

CD38 is highly expressed in inflammatory cells, including B cells, plasma cells, natural killer cells, dendritic cells, T cells, monocytes, macrophages, and neutrophils [1]. Amici et al. [8] showed that treating bacterial lipopolysaccharide (LPS) and IFN-γ in human macrophages and monocytes increased CD38 mRNA levels. Our studies were in accordance with Amici et al.’s results and showed that CD38 mRNA levels were enhanced by infection with an oral bacterial pathogen (*Pg* or *Aa*) or by stimulation with AGEs in murine BMMs (Figure 1A), supporting that CD38 is an inflammatory marker. However, we used live bacteria, which is biologically more relevant than LPS. Because CD38 degrades NAD^+^, we observed significantly reduction in NAD^+^ in murine macrophages infected with oral pathogens or stimulated with AGEs compared with controls (Figure 1B, *** *p* < 0.001). Inhibition of CD38 by 78c or knockdown of CD38 by the CD38 shRNA significantly enhanced NAD^+^ levels in murine macrophages infected with oral pathogens or stimulated with AGEs (Figure 1B and Figure 8B, * *p* < 0.05, ** *p* < 0.01, *** *p* < 0.001). Our study supports that inhibition of 78c is a good therapeutic strategy to increase NAD^+^ levels with bacterial infection or inflammation. 

Over 200 compounds can inhibit CD38 and can be classified as NAD analogs, flavonoids, or 4-amino-quinolines [12]. The 78c belongs to the 4-amino-quinolines family. Compared to flavonoids, which are competitive antagonists of CD38, 78c displays uncompetitive inhibition of CD38 [12]. Therefore, 78c is considered a “specific” inhibitor of CD38 [20]. Amici et al. [8] showed that rhein or apigenin (flavonoids with CD38 inhibitory activity) suppressed IL-6 and IL-12p40 when induced by LPS and IFN-γ. Treatment with apigenin or 78c also suppressed IL-1β, IL-6, and TNF levels induced by LPS in microglia or astrocytes [43]. However, it is unclear how CD38 regulates pro-inflammatory cytokine release induced by bacteria or LPS. 

In the current study, we demonstrated that 78c reduced IL-1β, IL-6, and TNF-α expressions induced by oral bacterial pathogens (*Pg* or *Aa*) or by AGEs (Figure 1C–E). Mechanistically, we demonstrated that treatment with 78c suppressed NFĸB, PI3K, and MAPKs (ERK, JNK, p38 MAPK) protein kinases induced by *Pg*, *Aa*, or AGEs (Figure 2, Figure 3 and Figure 4). It is worth noting that the effects of 78c on MAPKs were different among different stimuli. We observed no significant differences in p-JNK protein expression induced by *Pg* between cells treated with vehicle or 78c (Figure 2E). In contrast, 78c suppressed p-JNK induced by *Aa* or AGEs (Figure 3E and Figure 4E). This is because bacterial components can stimulate different TLRs. *Pg* mainly activates the TLR2 [44], while *Aa* mainly activates the TLR4 [27]. AGEs bind with receptors for AGE (RAGE), initiating immune responses, and the AGE–RAGE signaling cascade interacts with the TLR4 signaling pathway [26]. Activation of different TLRs and different signaling cascades contributes to the variation in the activation of NFĸB, PI3K, and MAPKs (ERK, JNK, p38 MAPK) protein kinases induced by *Pg*, *Aa*, or AGEs. In the current study, knockdown of CD38 by a CD38 shRNA only reduced IL-1β, IL-6, and TNF-α levels induced by AGEs, but had limited effects on cytokine levels induced by the oral pathogen *Pg* or *Aa* (Figure 8C–E). Our studies support that the anti-inflammatory response associated with CD38 limits on certain stimuli (such as AGEs), and 78c has some off-target effects in suppressing IL-1β, IL-6, and TNF-α levels induced by the oral pathogen *Pg* or *Aa*. 

Previously, there were controversial results associated with how CD38 regulates osteoclastogenesis. A CD38 monoclonal antibody (daratumumab, used in a clinical trial to treat patients with multiple myeloma [45]) suppressed osteoclastogenesis and bone resorption induced by RANKL in monocytes derived from patients with multiple myeloma [46]. In contrast, another study [47] demonstrated that murine bone marrow cells derived from CD38 knockout mice increased osteoclastogenesis induced by RANKL compared with wild-type control. In this study, we showed that treatment with the CD38 inhibitor (78c) suppressed osteoclastogenesis and bone resorption induced by RANKL (Figure 5). Mechanistically, we demonstrated that inhibition of CD38 by 78c reduced osteoclastogenic factors, including *Nfatc1*, *Ctsk*, *Acp5*, *Oscar*, *Ocstamp*, and *Dcstamp* induced by RANKL (Figure 6). Moreover, treatment with 78c suppressed podosome components (including PI3K, Pyk2, Src, F-actin, integrins, paxillin, and talin) induced by RANKL (Figure 7). Inhibition of podosome components by 78c subsequently suppressed cellular adhesion and fusion (osteoclastogenesis response) induced by RANKL. Additionally, activation of NF-ĸB and MAPKs also contribute to RANKL-induced osteoclastogenesis [48,49]. Because 78c inhibited NF-ĸB and MAPKs induced by oral pathogens or AGEs (Figure 2, Figure 3 and Figure 4), 78c could possibly also inhibit NF-ĸB and MAPKs induced by RANKL, leading to suppressing osteoclastogenesis. In contrast, the knockdown of CD38 by a CD38 shRNA enhanced osteoclastogenesis and bone resorption induced by RANKL (Figure 9), which is in accordance with the previous study [47], which showed that CD38 knockout bone marrow cells increased osteoclastogenesis compared with controls. Our results support the notion that 78c possesses some off-target effects in suppressing osteoclastogenesis induced by RANKL. 

This study had some limitations. Firstly, we only performed in vitro studies to determine the effects of a CD38 inhibitor (78c) or a CD38-specific shRNA on pro-inflammatory cytokine production induced by an oral pathogen (*Pg* or *Aa*) or by AGEs. Because the periodontal inflammatory response is influenced by multiple bacteria from gingival pockets and, in some cases, by multiple bacteria and comorbidities, such as diabetes, future studies need to determine if inhibition of CD38 by 78c or knockdown of CD38 by the CD38 shRNA could reduce IL-1β, IL-6, and TNF-α levels induced by multiple bacteria and AGEs. Secondly, we observed that knockdown of CD38 by the CD38 shRNA only reduced IL-1β, IL-6, and TNF-α levels induced by AGEs, with limited effects on cytokine levels induced by the oral pathogen *Pg* or *Aa* compared with controls (Figure 8C–E). It was not clear what the causes of this discrepancy among different stimuli were. Future studies need to determine if AGEs (but not oral pathogens) could stimulate Ca^2+^ signaling pathways and affect pro-inflammatory cytokine release. Thirdly, other inflammatory bone loss diseases, such as rheumatoid arthritis and systemic lupus erythematosus, are involved in autoimmune responses [13,50]. A previous study [51] showed that treatment with the CD38 monoclonal antibody (daratumumab) in multiple myeloma patients reduced autoantibodies in five out of six patients. CD38 is highly expressed in autoantibody-producing plasma cells (PCs), and PCs play a key role in the generation of autoantibodies. It has been shown that PCs were reduced in multiple myeloma patients treated with daratumumab via its cytotoxicity to autoreactive PCs [51]. Additionally, another study [52] demonstrated that treatment with 78c in collagen-induced arthritis mice suppressed joint inflammation, reduced the levels of B cells, IL-6, and TNF-α, and increased the level of IL-10, energy metabolism, and spontaneous movement in mice. Future studies need to determine if the inhibition of CD38 by 78c could reduce autoantibodies in collagen-induced arthritis mice and if treatment with 78c could alleviate bone loss in animals with arthritis or periodontitis. 

## 5. Conclusions

As the NAD^+^ level is reduced in humans with various pathological conditions, including diabetes, inflammation, aging, rheumatoid arthritis, and neurodegenerative disorders [1,9,10,11,12], the inhibition of CD38 has become a therapeutic strategy for treating these pathological conditions [1,3,4,5,13]. Treatment with apigenin (a flavonoid with CD38 inhibitory activity) in obese mice increased NAD^+^ levels in the liver, reduced fasting blood glucose, decreased total triglycerides in the liver, and improved glucose tolerance, compared with control vehicle treatment [53]. Treatment with 78c in aged mice reversed age-related NAD^+^ decline [54] and increased the lifespan and healthspan of naturally aged mice [55]. Treatment with 78c improved several physiological and metabolic aging parameters, including glucose tolerance, muscle function, exercise capacity, and cardiac function in mouse natural and accelerated aging models [54,55]. Treatment with 78c in collagen-induced arthritis mice also alleviated joint inflammation [52]. In this study, we further demonstrated that the inhibition of CD38 by 78c displayed both an anti-inflammatory effect in response to oral pathogens and AGE stimulation and anti-osteoclastogenic properties in response to RANKL stimulation, suggesting that inhibition of CD38 by 78c could become a promising therapeutic strategy for treating periodontal diseases. Although standard non-surgical periodontal treatment and supportive periodontal maintenance using scaling and root surface debridement continue to be the “gold-standard” treatment for stage I–III periodontitis, there are still patients or sites that show poor response to non-surgical periodontal treatment and long-term supportive maintenance efforts. This could be due to sustained dysbiosis, bacteria invasion to periodontal tissues, or a non-resolving chronic inflammatory response. Therefore, the 78c could serve as an adjunctive therapy to alleviate periodontal inflammation and reduce alveolar bone loss. Especially for patients with diabetes, 78c can alleviate AGE-induced inflammatory response, enhance NAD^+^, and subsequently correct the defect cell metabolism in patients with diabetes, which conventional scaling and root surface debridement cannot achieve.

## Data Availability

The data presented in this study are available upon request from the corresponding author.
